# GNSS Radio Frequency Interference Monitoring from LEO Satellites: An In-Laboratory Prototype

**DOI:** 10.3390/s24020508

**Published:** 2024-01-13

**Authors:** Micaela Troglia Gamba, Brendan David Polidori, Alex Minetto, Fabio Dovis, Emilio Banfi, Fabrizio Dominici

**Affiliations:** 1 LINKS Foundation, 10138 Turin, Italy; micaela.trogliagamba@linksfoundation.com (M.T.G.); brendandavid.polidori@linksfoundation.com (B.D.P.); 2 Department of Electronics and Telecommunications (DET), Politecnico di Torino, 10129 Turin, Italy; fabio.dovis@polito.it; 3 Italspazio S.r.l., San Giovanni La Punta, 95037 Catania, Italy; e.banfi@italspazio.com

**Keywords:** GNSS radio frequency interference, jamming, spoofing, software, embedded platform, advanced RISC machine (ARM) processors, LEO

## Abstract

The disruptive effect of radio frequency interference (RFI) on global navigation satellite system (GNSS) signals is well known, and in the last four decades, many have been investigated as countermeasures. Recently, low-Earth orbit (LEO) satellites have been looked at as a good opportunity for GNSS RFI monitoring, and the last five years have seen the proliferation of many commercial and academic initiatives. In this context, this paper proposes a new spaceborne system to detect, classify, and localize terrestrial GNSS RFI signals, particularly jamming and spoofing, for civil use. This paper presents the implementation of the RFI detection software module to be hosted on a nanosatellite. The whole development work is described, including the selection of both the target platform and the algorithms, the implementation, the detection performance evaluation, and the computational load analysis. Two are the implemented RFI detectors: the chi-square goodness-of-fit (GoF) algorithm for non-GNSS-like interference, e.g., chirp jamming, and the snapshot acquisition for GNSS-like interference, e.g., spoofing. Preliminary testing results in the presence of jamming and spoofing signals reveal promising detection capability in terms of sensitivity and highlight room to optimize the computational load, particularly for the snapshot-acquisition-based RFI detector.

## 1. Introduction

The vulnerabilities of global navigation satellite system (GNSS) signals to radio frequency interference (RFI) are well known [1,2]. Moreover, the use of GNSS in civil, industrial, scientific, and military domains makes it a pervasive technology, and many applications—from communication networks synchronization, financial transactions, intelligent transportation systems, location-based services, to spaceborne orbit determination—rely on it as a service [3]. As a consequence, awareness about the risk posed by the intentional RFI to GNSS on many non-GNSS applications in the so-called domino effect is nowadays much more than consolidated [4,5]. Unsurprisingly, several newspaper and public reports, e.g., [6,7,8,9,10,11,12], to cite a few, witness incidents due to the two major threats, namely jamming and spoofing.

Jamming mainly relies on power and spectral occupation to deny the GNSS signals, whereas spoofing consists of faking GNSS-like signals with the final purpose of deceiving the state estimation of victim receivers. In addition to this primary categorization, intentional RFI can be further classified as *non-GNSS-like*, e.g., chirp jamming, and *GNSS-like*, e.g., spoofing and matched code interferers [2]. The interest of the scientific community in investigating countermeasures dates back more than thirty years. A comprehensive survey of intentional RFI and countermeasures, including detection, localization, classification, and mitigation, over the last four decades can be found in [13]. For an overview over the last decade, the interested reader can refer to [13,14,15,16,17] for spoofing and [18,19,20,21] on jamming.

Most of the methods presented in the aforementioned specialized literature on satellite navigation apply to terrestrial receivers, and only recently have researchers started looking at space as a good opportunity for GNSS RFI monitoring. Low-Earth orbit (LEO) satellites in particular offer a privileged point of view on Earth, allowing them to simultaneously track authentic GNSS signals and sense possible RFI emitters from the ground. Global coverage, offered by a higher number of satellites frequent refresh rates due to their orbital velocity, and the possibility of performing interfering event captures with a precise GNSS timestamp provided by LEO satellites are the main benefits of LEO-based receivers. Recent works also demonstrated their effectiveness as an augmentation of the current generation of medium-Earth orbit (MEO) GNSS satellites [22,23]

Historically, spaceborne GNSS sensors have been mainly developed for remote sensing purposes [24,25,26], e.g., radio occultation and reflectometry. In the last couple of decades, GNSS has been considered to support LEO space missions such as NASA’s Gravity Recovery and Climate Experiment (GRACE) mission and other programs [27]. Actually, there are plenty of works, such as [28,29,30,31,32,33], documenting RFI detection, characterization, and mitigation strategies in such applications. Most of them are not focused on GNSS RFI, e.g., [28,29,30,31] and/or mainly aim at determining their negative impact on GNSS-derived measurements, such as atmospheric and climate metrics, e.g., [32,33]. It is only in the past five years that GNSS RFI monitoring has captured the attention of both commercial and academic initiatives. On the commercial side, HawkEye 360 Inc. is the first US commercial company to claim the use of a satellite constellation to generate RF data analytics [34,35]. Under the boost of the US Department of Defense pilot program launched in 2022, many other companies, e.g., Spire Global, Inc.-San Francisco (CA), U.S. and Kleos Space S.A.-Luxembourg (LU), EU, started building their own businesses on satellite-based RFI geolocation [36]. On the academic side, GNSS RFI detection, classification, and localization from LEO have been demonstrated to provide promising results [37,38,39,40], i.e., authors successfully geolocated some real-world jamming and spoofing signals using data from the FOTON receiver mounted aboard the International Space Station (ISS) in [38,39] and two STRATOS satellites provided by Spire Global Inc. in [40]. Anyway, both the FOTON receiver and the STRATOS satellites were originally designed for GNSS radio occultation purposes. Although they can be repurposed for emitter location, they suffer from some limitations. For instance, in the raw signal sample recording system, collecting 2-bit quantized intermediate frequency (IF) samples with a sampling rate of around 6 Msps and 3 MHz double-sided RF bandwidth on GPS L1 and L2. Now, the low sampling frequency and low quantization depth have the clear advantage of reducing the amount of data to be stored and down-streamed to the ground segment. On the other hand, the narrow RF band is incompatible with modern wideband signals, such as the Galileo E5 a/b OS signals, which require a minimum bandwidth (double-sided) of at least 20 MHz [41]. Additionally, it is known that in order to implement advanced detection (and mitigation) techniques, a higher number of quantization bits is recommended [1,2,3,4,5,6,7,8,9,10,11,12,13,14,15,16,17,18,19,20,21,22,23,24,25,26,27,28,29,30,31,32,33,34,35,36,37,38,39,40,41,42,43]. High-bit-count analog-to-digital converters (ADCs) (at least 4 quantization bits) allow for a higher fidelity of the recorded signal; consequently, improved performance on classification and localization is also expected [43,44,45].

In this context, this paper presents a new spaceborne GNSS interference monitoring system for civil use and provides a warning protection service to the users. The system is specifically designed to detect, classify, and localize terrestrial GNSS RFI signals, particularly jamming and spoofing, on two bandwidths, i.e., GPS L1/Galileo E1 and GPS L5/Galileo E5a, and to provide an alert service to the GNSS user community. It is made up of two main components: the satellite on-board payload and the ground station. The former is in charge of performing early and quick RFI detection by collecting and inspecting raw GNSS signal samples, which are then forwarded to the latter for a detailed analysis, including classification and localization. This paper presents the software (SW) implementation of the RFI detection module on a breadboard to be hosted on-board a LEO satellite, from the selection of both the target platform and detection algorithms to their performance evaluation.

The paper is organized as follows: A short overview of the GNSS RFI monitoring system is sketched in Section 2, whereas the theoretical background for RFI detection, classification, and localization techniques and the preliminary algorithmic choices are discussed in Section 3. Section 4 describes the development of RFI detection SW module, whose preliminary performance evaluation and computational load analysis are detailed, respectively, in Section 5 and Section 6. The results are critically discussed in Section 7. Finally, conclusions are drawn in Section 8.

## 2. Overview of the GNSS RFI Monitoring System

**Figure 1:**
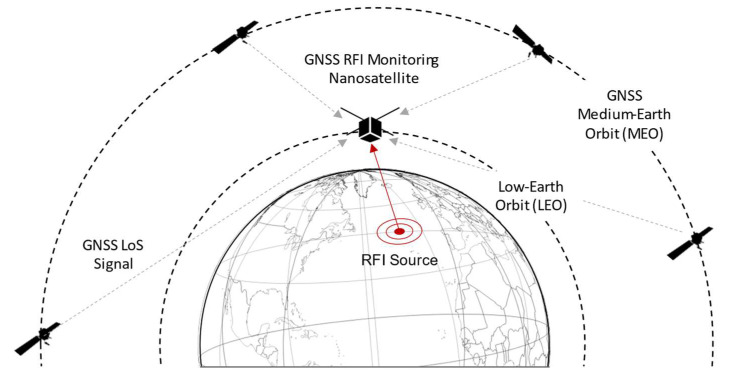
GNSS RFI monitoring system operational scenario (not to scale).

The system includes a space component and a ground component. The space component is essentially composed of the satellite payload. The payload, via a Nadir antenna oriented toward the Earth surface, is capable of receiving potential GNSS interference generated on the ground, as shown in Figure 1. Such a Nadir antenna is right-hand polarized (RHCP) in order to be able to receive any GNSS spoofing signal that might come from the ground while filtering legitimate GNSS signals that might be backscattered by the Earth surface. Furthermore, although the mission design has not been finalized yet, a simplified geometry for the coverage has been assumed. In this scenario, it can be assumed that only the noise is impinging on the Nadir antenna in nominal conditions (interference-free case), and the experiments have been conducted according to this assumption.

The whole payload is designed according to a software-defined approach, acting as a data grabber of raw Intermediate Frequency (IF) samples captured in L1-E1 and L5a-E5a bandwidths. Once the onboard receiving unit detects interference in the raw samples, it enables their storage as a binary file for further postprocessing at the ground station in order to validate the detection of the event and perform a classification and localization of the threat.

The overall antenna system is different w.r.t. the RFI detection proposed by [38] on ISS, which requires discriminating between legitimate and spoofing signals received at the same antenna.

The two main functional modules of the satellite payload are the Zenith and Nadir chains. The former includes a high-end GNSS receiver connected to a precise orbit determination (POD) Zenith antenna for on-board navigation solution computation and precise time source, and a GPS-disciplined oscillator (GPSDO) for ultra-stable clock generation and data time stamping. The latter consists of a radio frequency (RF) front-end (FE) in charge of receiving the Nadir L-band high gain antenna signal and spliting it into two sub-bands to be sent to the processing unit. Such a processing unit is responsible for (i) baseband downconversion and digitalization, (ii) GNSS RFI detection, (iii) storage of the raw samples, and (iv) interface with the satellite platform for command, telemetry, and data downlink. Finally, the ground component consists of a ground station(s) for mission control and data downlink and a ground processor to generate the service products, including the classification and localization of the GNSS interference captured by the satellites.

## 3. Theoretical Background

The detection, classification, and localization of terrestrial RFI sources by means of LEO satellites entangle the aims of the proposed GNSS RFI monitoring system. By addressing the satellite-based nature of this study, the aforementioned tasks can be described by the following item list.

- By *detection*, we refer to the capability of the satellite on-board system to detect RFI sources on Earth. RFI detection constitutes the trigger for the subsequent classification and localization.
- Once an RFI is detected, feature extraction allows for discriminating its nature. *Classification* refers indeed to those algorithms that are able to discriminate the received interference according to pre-defined classes based on specific signal features. In this regard, researchers have recently applied artificial intelligence (AI)/machine learning (ML) algorithms to classify interference: we can find examples of this in [46,47,48,49,50,51]. Convolutional neural network (CNN) architecture, commonly used for image classification, can be applied to this problem. As an example, in [46] the authors show the improvement of the levels of accuracy reachable with a feature-aided CNN with respect to a baseline CNN.
- *Localization* covers aspects related to the techniques conceived to pinpoint the position of interference sources on Earth. RFI source localization has been extensively studied in the field of radar and remote sensing technologies, and consolidated techniques can be used in the presence of both single and multiple satellites observing RFI emission [52]. Well-known techniques for emitter localization are based on time-of-arrival (ToA) and frequency-of-arrival (FoA) that foresee the estimation of the signal propagation time and the carrier frequency. However, unknown signals transmitted over unknown carrier frequencies are typically localized through their differential counterparts, i.e., time-difference-of-arrival (TDoA) and frequency-difference-of-arrival (FDoA). When multiple antennas are available at the observer, angle-of-arrival (AoA) and differential AoA (DAoA) can be considered. Based on directional measurements regarding specific spatial regions, received signal strength (RSS) can be considered to determine RFI emitters.

In the following subsection, only the fundamental aspects of RFI *detection* in GNSS signals are recalled, as they constitute the main theoretical background for the development of the payload detector described in this manuscript. Although RFI classification and localization have also been addressed, the implementation of those algorithms is deemed out of the scope of this article.

### 3.1. RFI Detection in GNSS Receivers

For monitoring purposes, we can categorize RFI detection techniques based on a generalized GNSS receiver architecture. Its main functional blocks can be identified as (i) front end assembly (i.e., antenna and ADC), after which potential RFI can be inspected through dedicated pre-correlation techniques; (ii) the acquisition stage and tracking loops that produce metrics suitable for the so-called post-correlation techniques; and eventually, (iii) the navigation algorithm engine that is responsible for inferring the receiver state vector, i.e., position, velocity, local clock offset, and drift. As highlighted, the most popular and effective solutions for RFI detection in GNSS mainly refer to pre-correlation and post-correlation techniques. Some examples are provided hereafter for the sake of completeness, while RFI detection strategies performed at the navigation algorithm engine are not mentioned. This is because RFI typically undermines the reliability of the PVT output solutions and hinders their use for prompt and effective detection.

At the front-end stage, a popular technique for RFI detection is automatic gain control (AGC) monitoring, documented in [53,54,55,56]. The AGC is designed to adapt the received signal dynamics to the environmental conditions in which the receiver is located. A numerical control amplifier is in charge of providing gain or attenuation to the incoming signal to keep the signal amplitude suitable for the subsequent analog-to-digital conversion. As a result, it can serve as a tool for investigating unexpected dynamics and fluctuations of the incoming signal power due to interferences. The dependence on the ambient temperature and the hardware components of the receiver implies that ACG monitoring requires calibration. This calibration consists of empirically defining an interference detection threshold, also based on the averaged value of the AGC. In [56], the authors demonstrated the ability to detect wideband interference using AGC monitoring up to a jamming over signal ratio of $\frac{J}{S}=31\;\mathrm{dB}$, with J being the jammer’s power level and S the legitimate signals’ level. This technique was discarded for three main reasons. The first being that the minimum detectable power using other techniques is higher; secondly, the detailed hardware characteristics of the system are yet to be defined, given that this technique is highly dependent on the system implementation, thus imposing a non-negligible limitation. Finally, in other techniques, the AGC needs to be disabled, which would lead to obstacles.

Next in the receiver chain, we find pre-correlation techniques. These operate on the raw in-phase and quadrature signal samples after the AGC and analog-to-digital converter (ADC).

Among the pre-correlation techniques, an effective method is the frequency power detector (FPD). This method is based on the spectral estimation of the non-stationary signal in the presence of noise. The metric used for the test statistic is the estimated spectrum of the signal obtained by processing *N* measured samples. The quality of the spectral estimation depends on multiple factors, and being an estimator, it is characterized by an intrinsic variance, which becomes a source of uncertainty impacting the detection performance. In this work, the version implemented is based on a technique proposed in [1], where the short-term Fourier transform (STFT) is leveraged to pursue time-frequency analysis. The interference-free spectral estimate can be assumed to be known after proper calibration of the antenna and RF front-end setup. As an example, using the 1 ms detection window, the detection probability is higher for a front-end filter with a 20 MHz bandwidth, while a 5 MHz wide front-end enhances the false interference detection probability due to the reduced number of samples used to estimate the spectrogram, which increases the standard deviation of the spectral estimation. Nevertheless, the analysis window is sufficiently large to enable wideband interference detection even for interference power lower than J = −142 dBW. For the 1 µs detection window, the detection probability is higher for a front-end filter with a 20 MHz bandwidth, and no relevant differences are observed compared to the case at the 1 ms analysis window. For a front-end bandwidth of 5 MHz, the false interference detection probability is higher, and larger observation widows are recommended to achieve higher detection performances.

Another pre-correlation technique explored is the chi-square goodness-of-fit (GoF), based on the known legitimate signal’s sample probability density function. The algorithm relies on the fact that in the nominal case, i.e., when no interference is present, only the thermal noise dominates (the GNSS signal is buried in the noise), and the raw samples out of the ADC are normally distributed random variables with a certain mean and a certain variance ($H_{0}$ hypothesis, i.e., absence of RFI) [57]. On the other hand, in the presence of an interfering signal, raw sample distribution significantly differs because of the induced distortion ($H_{1}$ hypothesis, i.e., presence of RFI). Based on these assumptions, the GoF is able to estimate how much the two distributions differ by means of a statistical metric, the so-called *p*-value, which is the probability that the two distributions have the same statistical characteristics. When no disturbances affect the signals, the *p*-value is close to one. On the contrary, in a critical scenario where interference is present, the *p*-value assumes smaller values. A threshold mechanism is used to decide on the binary hypothesis, set on the basis of the ‘significance level’ of the test. For a thorough theoretical description of the GoF statistical test, the reader can refer to [57,58].

In [56], the Chi-square GoF method was used to detect the presence of both continuous wave and wideband interference to the minimum level of jammer-to-signal ratio of $\frac{J}{S}=23\;dB$. One of the limitations of this technique regards the filter bandwidth, which, when smaller than the jammer’s bandwidth, prevents the receiver from gathering the full jamming power. On the other side, beneficial aspects are that it does not require a priori information on the interference signal characteristics, and the computational burden is rather low since no complex algorithm other than the evaluation of the histograms is needed. The results of the simulation campaign depict the success ratio of the detection for received jammer power at the LEO satellite in the range $P_{j}\in \;[-139,-151]\;dBW$, while meeting system complexity requirements in the definition of a low-cost payload.

In post-correlation techniques, we find post-correlation observations such as power distortion monitoring, multi-correlator banks, and signal quality monitoring (SQM). They all rely on the output of the correlators in the tracking stage. Even if they show high potential for interference monitoring since they detect the distortions of the GNSS signal due to the presence of the interference, they are not fully applicable here, where the GNSS is not present and only RFI is received. Post-correlation techniques are mostly applicable in reflectometry and radio occultation scenarios, which differ from the scenario under investigation.

Another approach to dealing with interference detection (and, if needed, classification/mitigation) is based on the use of advanced signal processing techniques that allow the representation of the signal digitized by the ADC of the receiver, in a different domain, where the information related to the interference can be better identified, isolated, processed, or mitigated. These algorithms are referred to as transformed domain (TD) techniques. Among others, we find the wavelet packet decomposition (WPD) and the Karhunen-Loève transform (KLT). These methods are further explained in [1]. As examined in [59], transformed domain techniques show great potential for mitigation purposes, while they are more computationally complex. WPD can be implemented through digital filter banks, while KLT requires high computational loads. In [13], transformed domain techniques are mostly seen as mitigation algorithms and are discussed as such. However, such techniques are mainly oriented toward the mitigation of the RFI effects on GNSS receivers, a task that falls outside the goal of this research.

## 4. Development Work

### 4.1. Selection of the Target Platform

The choice of a platform suited for the implementation of the RFI detectors was driven by the following criterion: it should include (i) a RF front-end (FE) for the baseband (or intermediate frequency) down-conversion and digitalization and (ii) a processor assisted by a hardware accelerator, e.g., a field programmable gate array (FPGA), for the most demanding operations.

According to this, the platform originally selected was the Analog Devices ADRV9361-Z7035 [60]. Such a platform is made of a configurable RFFE, namely the AD9361, and a Xilinx Z7035 Zynq-7000 All Programmable System-on-Chip (SoC), which is based on a Dual-Core ARM Cortex-A9 processor and a Xilinx Kintex-7 FPGA. To ease its use, ADRV9361-Z7035 can be mounted on the carrier board ADRV1CRR-FMC [61]. The manufacturer provides reference designs for both the FPGA and the ARM processor, along with a library, namely the libiio [62], developed by Analog Devices to ease the development of software interfacing Linux Industrial I/O (IIO) devices. It can be noticed that components analogous to these ones are available in commercial products already qualified for use in space applications, e.g., [63]; this could foster the transition from the prototype to the final product.

Unfortunately, in 2021, when the work started, the ADRV9361-Z7035 needed a revision due to the scarcity of available components on the market, and it was not available for purchase in the short term. The final choice then was the Analog Devices Adalm Pluto [64,65], which includes the Analog Devices AD9363 RFFE and the Xilinx Zynq Z7010 SoC. Due to its characteristics, Adalm Pluto can be considered a low-profile version of ADRV9361-Z7035, thus granting the compatibility of the implemented designs with the more expensive and performing board.

### 4.2. Implementation of the RFI Detectors

On the basis of the overview and analysis reported in Section 3.1, two algorithms have been selected for RFI detection: one for *non-GNSS-like* interference, e.g., chirp jamming, and the other for *GNSS-like* interference, e.g., spoofing. The chi-square GoF algorithm is used in the former case, whereas the latter is tackled by a snapshot acquisition of GPS and Galileo signals. The GoF detects the presence of interferences that change the statistical characteristics of the incoming signal, whereas the snapshot acquisition provides a rough Doppler-delay estimate, thus detecting interferences that are fake GNSS-like signals. In this regard, the choice of the latter is endorsed by the application context: from space, spoofing signals can be detected at the earliest stage of the GNSS elaboration chain, namely the acquisition stage, whereas this is not possible on a terrestrial receiver where the acquisition cannot distinguish the true GNSS signal from a potentially fake one.

#### 4.2.1. Chi-Square Goodness of Fit (GoF) Test

The chi-square GoF test [58], as explained in Section 3.1, acts as a pre-correlation monitoring point along the receiving chain. It is based on a test statistic for monitoring the distortion of the distribution of the raw samples out of the FE’s ADC.

The GoF algorithm is implemented in C language and is executed by an Intel processor-based laptop computer: the executable connects with the AD Adalm Pluto board through the AD library libiio, configures the Adalm-Pluto’s RF FE, and receives and elaborates the samples. The same source code has been cross-compiled for ARM processor and executed directly on the Adalm-Pluto embedded processor. In the current implementation, the algorithm performs the following steps:

1. Save a snapshot of the incoming samples;
2. Compute the histogram of the samples in the snapshot, namely the occurrences of the samples’ values;
3. Compare the current histogram with a reference one, evaluating the *p*-value;
4. Declare the presence of the interference in case the *p*-value is below the threshold, the so-called significance level.

This sequence of operations is performed continuously at a certain rate, e.g., 1 Hz by default, which is configurable by the user. The reference histogram for the evaluation is obtained during the calibration phase, for which different configurations can be selected by the user. By default, the first snapshots of samples are used to get the reference histogram. Alternatively, the user can choose to load a pre-calculated reference histogram from an input file. Moreover, the same reference histogram can be used for the whole execution, or it can be updated regularly. In this last case, the GoF continuously refreshes the reference histogram using the incoming samples.

In order to keep the computational load as low as possible, the histograms are evaluated through the use of a lookup table (LUT), avoiding the use of assembly and/or FPGA resources. The output of the GOF is a binary detection flag, which indicates the absence (value 0) or the presence (value 1) of an interference.

An important aspect to be considered when configuring the AD Adalm Pluto FE is that the GoF requires the AGC set to work in manual mode with a fixed gain in order to build the reference histogram. Thus, an initial AGC calibration in nominal conditions has to be performed to find the optimal gain so that the histogram exhibits a Gaussian distribution shape and fully exploits the whole ADC dynamic, namely all 12 available bits.

#### 4.2.2. The Snapshot Acquisition

A classical fast-Fourier-transform-(FFT)-based parallel code-phase search (PCS) algorithm [66], employed to acquire GPS and Galileo signals in NGene software receivers’ series [67,68,69], has been identified as a good starting point.

**Figure 2:**
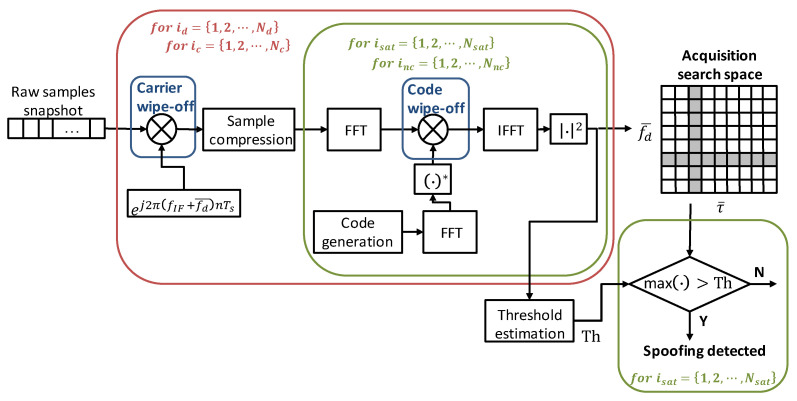
Block diagram of the FFT-based parallel code-phase search algorithm used as GNSS-like RFI detector. The $\left(\cdot \right)\;$operator indicates the complex conjugate.

The software routines have been properly modified to elaborate a snapshot of samples, adapt them to cope with the Doppler dynamics of LEO satellites, and optimize them to reduce the computational load as much as possible. It is in fact known that acquisition is one of the heaviest functions from a computational standpoint in any conventional GNSS receiver. The primary functional units and operations are illustrated in Figure 2. At first, a snapshot of the raw samples is saved, and then the algorithm performs a search of the whole set of GPS and Galileo satellites. Once the nominal carrier has been removed, an averaging operation, also indicated as sample compression in Figure 2, is performed to compress 1 ms of samples to fit the number of FFT points. After that, the code wipe-off takes place, multiplying the input sample FFT with the FFT complex conjugate of the locally generated PRN code, and the IFFT and square modulus are applied. Four nested loops group the above-described operations: such loops are determined by the acquisition parameters $N_{d}$, $N_{c}$, $N_{sat}$, and $N_{nc}$, which represent the number of searched Doppler bins, code delay bins, satellites, and non-coherent sums, respectively. Finally, for each satellite, if the maximum exceeds the threshold, the satellite acquisition is declared, i.e., a GNSS-like RFI signal (spoofing or matched-code interferer) is detected.

The main differences with respect to [67] regard the granularity of the operations. Indeed, in the original algorithm, the acquisition is performed for one satellite at a time, elaborating contiguous 1 ms bunches of samples. In the modified one, the acquisition is executed on non-contiguous snapshots of raw samples, e.g., the first 8 ms of samples every second, searching for all satellites of the GPS and Galileo constellations. Carrier (and subcarrier for Galileo) wipe-off, sample compression, and threshold computation are repeated for each PRN in the former algorithm, while they are performed just once for the whole snapshot and all satellites of the same constellation. The threshold, in particular, is estimated for the first PRN of the constellation and used for all other satellites in the same constellation. Finally, some scaling parameters used in the FFT and IFFT functions have also been properly tuned to fit the dynamics of the input signal.

The software routines have been first implemented on a standard PC and then ported to Adalm-Pluto’s processor. It is worth noticing that most of the blocks in Figure 2, such as carrier generation, wipe-off operation, FFT, and IFFT, have to process high data rates, i.e., samples coming from the FE at several MHz, thus being coded in assembly language, exploiting processor-specific optimizations. Such optimizations grant real-time performances; the samples coming from Adalm-Pluto ADC are elaborated on the fly.

#### 4.2.3. The RFI Detection Software Module: A General Overview

The main functionalities of the final RFI detection software module can be summarized as follows:

1. The detection of *non-GNSS-like* signals and *GNSS-like* RFIs by means of the GoF and the FFT-based PCS snapshot acquisition, respectively;
2. The elaboration on the fly of snapshots of raw samples out of the ADC;
3. The generation of a sinusoidal tone used to up-convert the L5/E5 signal at $f_{L5}=1.17645\;GHz$ to the L1/E1 band at $f_{L1}=1.57542\;GHz$, as better described in the next section;

Furthermore, it exploits the software-defined-radio (SDR) approach, granting the highest level of flexibility, configurability, maintainability, and portability of a fully software implementation. Two versions are currently available: the former tailored to Intel x86 processors and the latter to ARM-based ones. The interfaces to the LEO satellite on-board computer have also been defined; in the current version, the configuration parameters and outputs are respectively read from and logged into files.

It is worth noticing that the RFI detection software module works for the GPS L1/Galileo E1. Concerning the additional required bandwidth, i.e., the GPS L5/Galileo E5a signals, the GoF is directly compatible, while the snapshot acquisition should be adapted. Indeed, analyzing the distributions of the raw samples out of the ADC, the GoF does not depend on the GNSS bandwidth, and it can directly work on another band without any algorithmic modification. On the other side, the snapshot acquisition, which is strictly dependent on the signal structure, including code rate, period, and length, is not directly compatible and should be modified. In this regard, the limitations of the Adalm-Pluto board in terms of computational resources and the number and configuration of RF receiving channels did not allow the implementation and testing of the L5/E5a detection, which has been left to future evolutions of the system. In particular, the first encountered limitation regarded the maximum RF bandwidth and sampling frequency supported, making the board not compatible with the L5/E5 band. In fact, at the required sampling frequency, i.e., at least 20 Msps, corresponding to 80 MBps, losses of raw samples occur, most likely due to the limit of the USB 2.0 stream speed. Furthermore, a cold-start acquisition on the L5/E5a signal is unfeasible due to the very high computational load [70]. The alternative approach, namely a L1/E1-aided acquisition [71], cannot be tested in the current version of the system since the Adalm-Pluto features only one receiving channel, so that the two GNSS bandwidths, i.e., L1/E1 and L5/E5a, cannot be received and elaborated in parallel.

## 5. Detection Performance Evaluation

**Figure 3:**
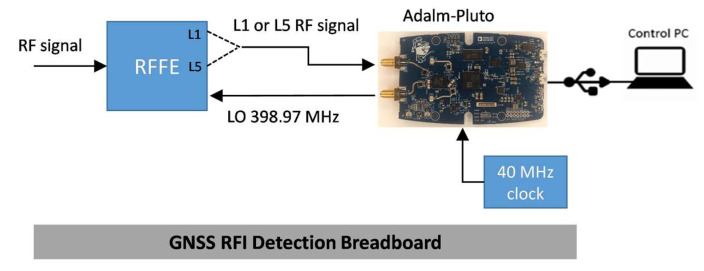
Block diagram of the GNSS RFI detection breadboard to be hosted on-board a LEO satellite.

In order to evaluate the performance of the RFI detection software module, a test campaign has been conducted, involving the whole GNSS RFI detection breadboard. As depicted in Figure 3, it is composed of two main devices: an RFFE specifically developed by Italspazio and the Adalm-Pluto board. The former is in charge of splitting the two GNSS RF bands, namely the L1/E1 and L5/E5, whereas the latter down-converts to baseband, digitalizes them, and hosts the RFI detection software module. For sake of clarity, the RFFE provides the L5/E5 signal upconverted to $f_{L1}=1.57542\;GHz$, using the sinusoidal tone produced by the Adalm-Pluto board and mentioned in Section 4.2.3. This is required for forward compatibility with the originally selected board, i.e., the ADRV9361-Z7035, that features two parallel receiving channels sharing the same local oscillator. The Adalm-Pluto FE, i.e., the AD9363, elaborates one RF channel at a time and requires an external 40 MHz clock source with higher stability than the aboard TCXO. The RFI detection software module runs on the ARM processor. Finally, a control PC is connected to the board via the USB port.

Two interference scenarios have been considered: jamming and spoofing. The former is used to assess the RFI detector based on the GoF, whereas the latter is employed for the snapshot acquisition. Two key performance indicators (KPIs) have been selected and listed as follows:

1. The sensitivity is defined as the minimum level of detected interfering power;
2. The detection capability, i.e., the capacity to properly activate/deactivate when the interference is present/absent (OFF-ON test).

All the tests are performed for the L1/E1 band with the Adalm-Pluto FE configured as reported in Table 1. As pointed out in Section 4.2.1, the AGC has been set to work in manual mode, as required by the GoF to build the reference histogram.

### 5.1. Jamming Detection Evaluation

**Figure 4:**
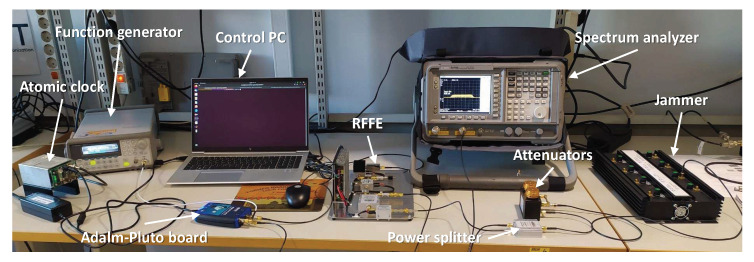
Test setup for the jamming detection evaluation.

Figure 4 shows a picture of the test bench prepared to evaluate the GoF-based RFI detector. A wideband real jammer is injected into the Italspazio’s RFFE with variable power by means of a chain of fix and variable attenuators and provided as input to the Adalm-Pluto FE configured as in Table 1. The Adalm-Pluto receives a stable 40 MHz external clock from a function generator, using a rubidium atomic clock as a reference, and it is connected to the control PC via a USB port. The spectrum analyzer provides an estimation of the jamming power.

As for the jamming scenario, a real wide- and multi-band chirp jammer has been selected since it is representative of a jammer family among the most frequently detected in the real world [72]. It is worth noticing that the GoF effectiveness on different kinds of interferences, including continuous wave (CW), narrowband (NB), and wideband (WB), has already been demonstrated in literature, e.g., in [58]. Furthermore, the algorithm detection capabilities mainly depend on the interfering power, independent of the structure of the jammer, e.g., NB, WB, linear/triangular/quadratic chirp, with no need to modify the internal parameters.

Table 2 lists the main GoF parameters used for the tests: the evaluation is performed every second using a snapshot of 10 ms and computing the histogram over 32 bins. A reference histogram file has been saved once when the jammer is switched off and used when the jammer is on for the whole duration of the test. The reference histogram is obtained averaging 10 histograms, each one computed every second using a snapshot of 10 ms in the absence of interference. Finally, the significance level has been set to 0.95.

**Figure 5:**
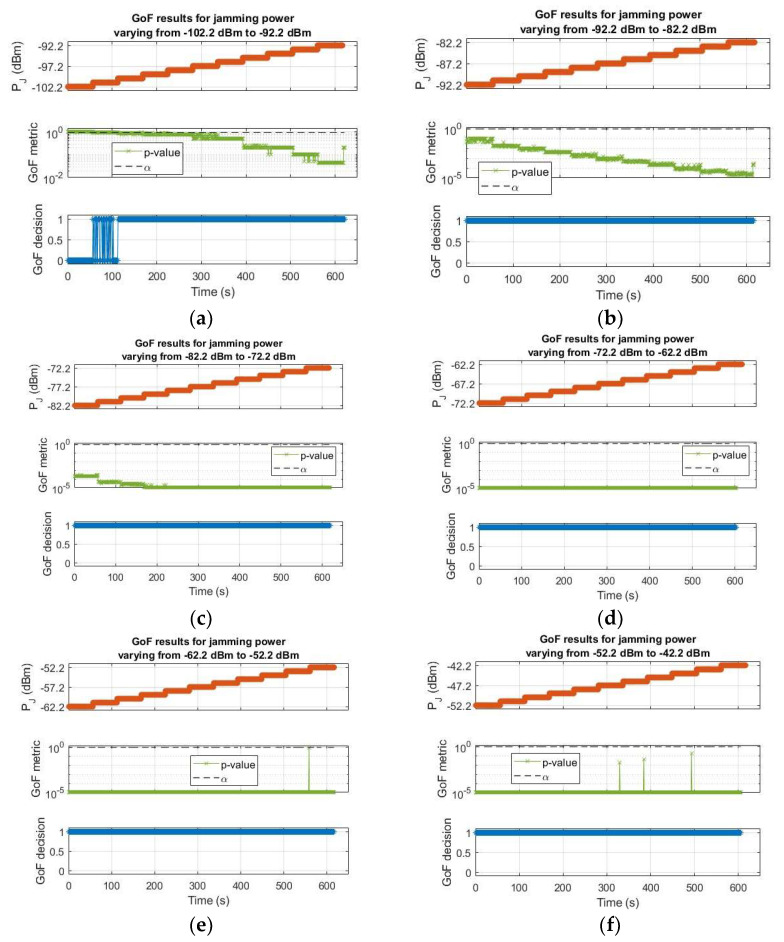
GoF-based RFI detector results for the sensitivity test: jamming power varying from −102.2 dBm to −92.2 dBm (**a**), −92.2 dBm to −82.2 dBm (**b**), −82.2 dBm to −72.2 dBm (**c**), −72.2 dBm to −62.2 dBm (**d**), −62.2 dBm to −52.2 dBm (**e**), and −52.2 dBm to −42.2 dBm (**f**).

**Figure 6:**
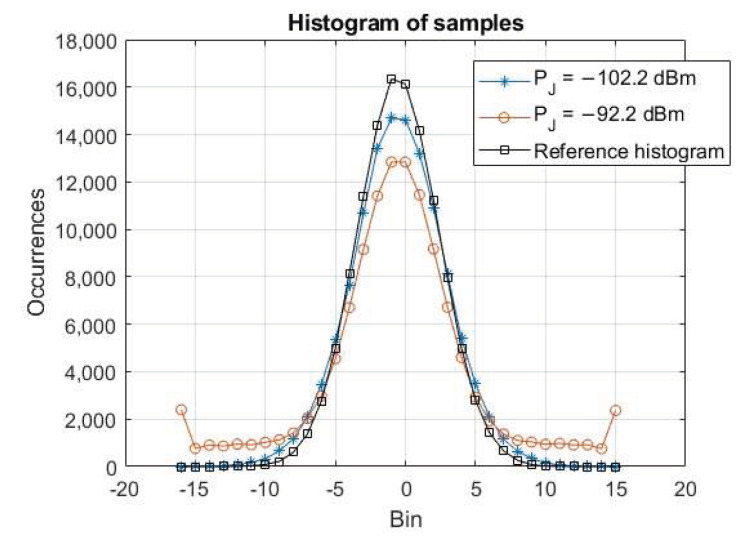
Histogram of the raw samples collected during the sensitivity test for two jamming power levels, i.e., −102.2 dBm and −92.2 dBm, and the reference histogram.

In order to assess the sensitivity, the jamming power has been varied from −102.2 dBm to −42.2 dBm with a step of −1 dB per minute for a total duration of the test of 1 h. Raw samples have been collected over windows of 10 min to ease the analysis and post-processed by the GoF-based RFI detector, namely the GoF. Figure 5 shows the achieved results: each figure reports the time evolution of the jamming power $P_{J}$ in the top plot and the GoF outputs, namely the *p*-value and the activation flag, respectively, in the middle and bottom plots (0 = no interference, 1 = interference detected). The threshold, namely the significance level α, has also been reported in the middle plot, where the logarithmic scale allows one to better appreciate the high sensitivity of the detector, which shows it is able to tightly follow the power variations. As visible in Figure 5a, the GoF always successfully detects jamming signals with a power higher than −100.2 dBm, preceded by a missed detection region at −101.2 dBm (about 50% of the missed detection rate). For very strong jamming power in Figure 5e,f, the *p*-values show some points with higher values (see the green line with the x marker), but still under the threshold. As exemplary cases, Figure 6 reports the histogram of the collected raw samples for two jamming power levels: −102.2 dBm, i.e., below the sensitivity, represented by the blue line with the stars marker, and −92.2 dBm, i.e., above the sensitivity, represented by the red line with the circle marker. It can be clearly seen that the difference with respect to the reference histogram (black line with square marker) is relevant in the latter case (detection), while it is very small in the former case (no detection). Obviously for higher jamming power, the distortion becomes even more evident.

**Figure 7:**
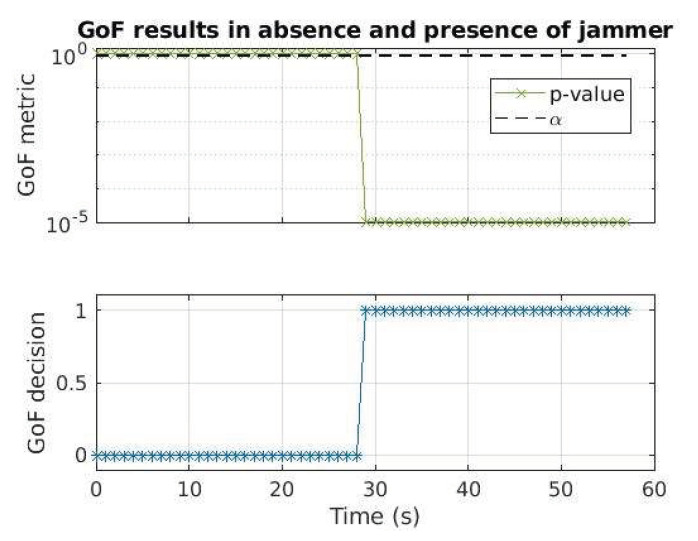
GoF-based RFI detector results for the OFF-ON test: the jammer is switched on around 29 s with −42.2 dBm power.

In order to assess the detection capability, a simple OFF-ON test is performed: the jammer is off, and then it is switched on around 29 s with a strong power, namely −42.2 dBm. As shown in Figure 7, the GoF correctly detects the jamming signal after it has been switched on, as expected.

### 5.2. Spoofing Detection Evaluation

**Figure 8:**
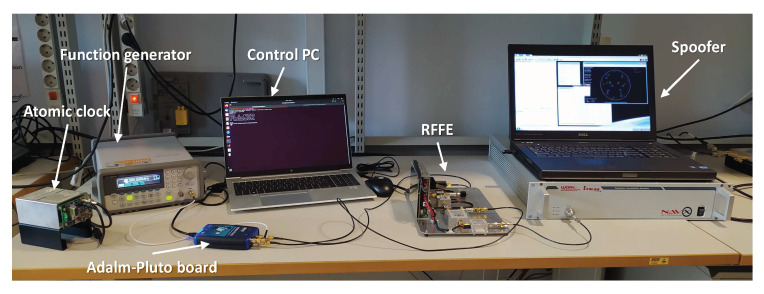
Test setup for the spoofing detection evaluation.

Figure 8 shows a picture of the test bench prepared to evaluate the RFI detector based on the snapshot acquisition. The test bench is similar to the one in Figure 4, but the jammer has been substituted with a spoofing signal generator. The spoofing signal is generated with the IFEN NAVX NCS GNSS simulator [73], injected into the Italspazio’s RFFE, and provided as input to the Adalm-Pluto FE configured as in Table 1.

The generated RF signal is saved to a file and post-processed by the snapshot acquisition, whose parameters are listed in Table 3 for both the GPS L1 and Galileo E1B. Five sets of values, i.e., from S1 to S5, have been evaluated for GPS, whereas four sets, i.e., from S6 to S9, have been considered for the Galileo. More in detail, the main parameter changed is the number of non-coherent sums, which represents the number of code periods of raw samples to be elaborated and consequently the snapshot length directly derived from that. The number of coherent sums is 1 code period, i.e., 1 ms for GPS and 4 ms for Galileo. Then, for both signals, the FFT size is set to 4096 complex points and the false detection probability is set to 1 × 10^−11^, whereas the other parameters define the size of the search space in the Doppler and code delay dimensions. The Doppler search space ranges in the ±20 kHz interval to cover the LEO dynamics, with a different step for GPS and Galileo depending on the cross-ambiguity function (CAF) shape. In this regard, for the Galileo acquisition, a couple of Doppler step values have been evaluated, as visible in the last four columns of Table 3.

**Figure 9:**
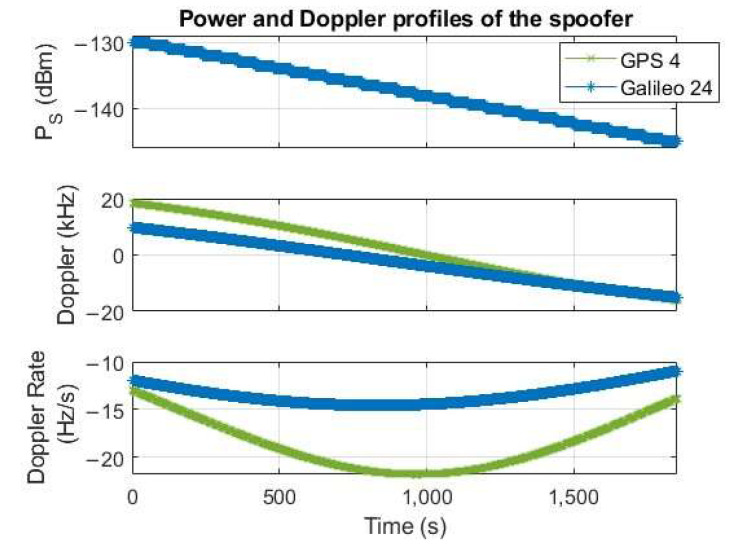
Power and Doppler profiles of the generated spoofing signals.

In order to assess the sensitivity, the NAVX NCS GNSS simulator has been properly set to generate 1 GPS L1 and 1 Galileo E1 signals, varying the satellite power from −130 dBm to −145 dBm with a step of −0.5 dB every minute for a total duration of the test of half an hour. For each satellite, the Doppler profile is plausible of a LEO orbiting satellite and the transmitting source in a static position on the Earth’s surface. Figure 9 reports the power $P_{S}$ and Doppler profiles of the generated signals, showing the typical range and dynamics of a LEO satellite.

It is worth remarking that, as mentioned in Section 4.2, the snapshot acquisition-based RFI detector targets *GNSS-like* interferences, including not only spoofing but also matched-spectrum or matched-code jammers, since they broadcast fake binary-code-modulated signals. Indeed, similarly to the GoF-based algorithm, the detection capability of the snapshot acquisition-based detector mainly depends on two factors: (i) the presence of GNSS spreading code(s) and (ii) the interfering power, independently of other internal features of the interference, e.g., content partially (matched-spectrum jammers) or mostly (spoofing) identical to an authentic signal, type of spoofing attack (asynchronous, synchronous, synchronous with multiple transmitters and meaconer attacks). According to this, the simulated scenario can be considered sufficiently representative of the target interference, at least as a first instance.

**Figure 10:**
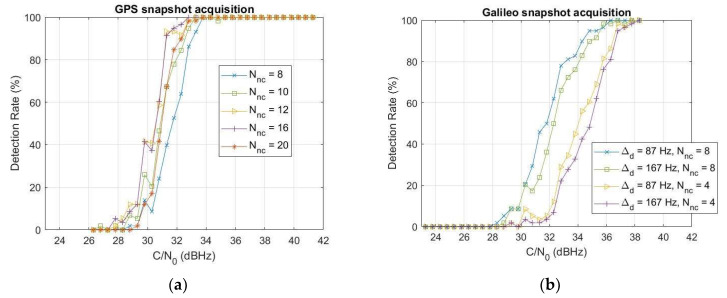
Detection rate of the GPS 4 (**a**) and Galileo 24 (**b**) as a function of the carrier-to-noise ratio, obtained respectively with the sets of parameters S1–S5 (different values of non-coherent sums $N_{nc}$) and sets of parameters S6–S9 (different values of non-coherent sums $N_{nc}$ and Doppler step ${∆}_{d}$) in Table 3.

In order to have an overall picture of the results, Figure 10 reports the detection rate as a function of the carrier-to-noise ratio $C/N_{0}$ and the different sets of parameters reported in Table 3 for both GPS L1 and Galileo E1B. The detection rate, also called activation percentage, is defined as the percentage of time in which the detector is correctly activated. For this analysis, a noise power spectral density $N_{0}=-201.3\;dBW/Hz$ is assumed. For both GPS and Galileo, we can observe an improvement in the detection rate as the number of non-coherent sums $N_{nc}$ increases, as expected. For GPS in Figure 10a, such improvement reaches a limit with $N_{nc}=\{12,16\}$, showing similar performance, then a worsening is clearly visible with $N_{nc}=20$. For Galileo in Figure 10b, the analysis is limited to $N_{nc}=8$, corresponding to a 32 ms snapshot. Considering the higher number of non-coherent sums and consequently longer snapshots implies a higher computational complexity and execution time, as also better analyzed in Section 6. The effect of the Doppler step ${∆}_{d}$ is not so relevant: performance for the two considered values, i.e., ${∆}_{d}=\left\{87,\;167\right\}\;Hz$, are close for the same number of non-coherent sums, with a difference in the order of 0.5 dB. The sensitivity for 95% detection probability is in the range 32–33.5 dBHz for GPS and 35.5–37 dBHz for Galileo, corresponding respectively to −139.3–−137.8 dBm and −135.8–−134.3 dBm.

**Figure 11:**
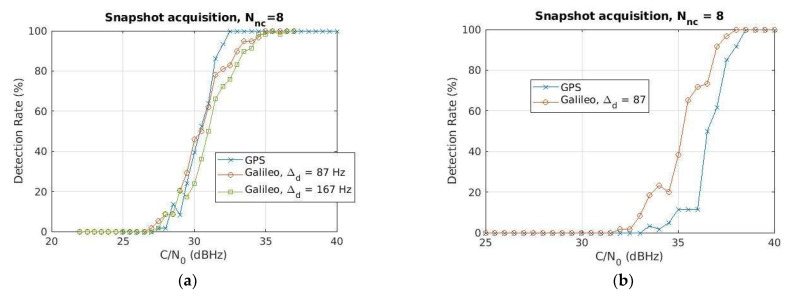
Comparison of the GPS L1 and Galileo E1B detection rate for the same number of non-coherent sums $N_{nc}=8$, and LEO’s compatible Doppler profile as in Figure 9 (**a**) and close-to-zero constant Doppler (**b**). In (**b**), the GNSS input signal has been generated with NFUELS [74] with constant Doppler equal to 50 Hz for both GPS and Galileo satellites.

Comparing the curves for the same $N_{nc}=8$, as reported in Figure 11a to ease the readability, the performance is comparable for medium-low% detection probabilities, particularly for the smaller Galileo Doppler step value, namely ${∆}_{d}=87\;Hz$ (see the red line with circle markers), whereas the GPS overcomes the Galileo with a sensitivity advantage of about 2 dBHz at 95% detection probability. Such an advantage can be likely explained considering the LEO’s Doppler dynamics, implying a shift of the acquisition peak and consequently a worsening of the performance for larger snapshots: $N_{nc}=8\;$ translates into an 8 ms snapshot for GPS but 32 ms for Galileo. Ideally, with zero or close to zero Doppler, GPS and Galileo performances are expected to be similar or even better for Galileo, as confirmed in Figure 11b, showing the detection rate comparison between GPS and Galileo for the same $N_{nc}=8\;$ (set of parameters S1 and S6 in Table 3, respectively) and Doppler close to zero. For sake of clarity, in this last experiment, the GNSS signal has been generated using a MATLAB-based GNSS simulator (N-FUELS [74]) with a different FE model. Although these results in Figure 11b cannot be directly compared with those achieved with the breadboard in Figure 11a, they are worth reporting since they confirm the acquisition peak shift effect due to the LEO’s Doppler.

**Figure 12:**
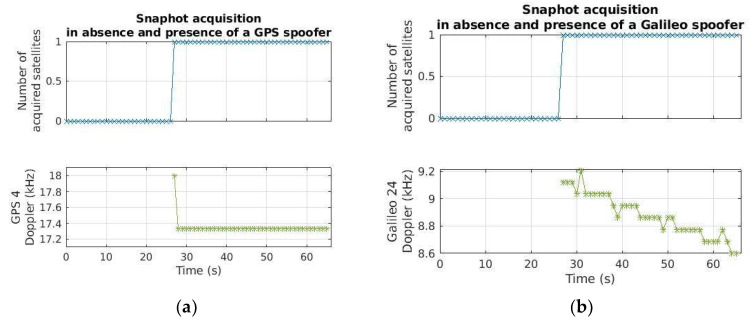
GPS L1 (**a**) and Galileo E1B (**b**) snapshot acquisition results, obtained with the sets of parameters S1 in Table 3 and Table 4, respectively, for the OFF-ON test: The spoofer is switched on around 27 s with −130 dBm power.

Regarding the detection capability, as for the GoF, a simple OFF-ON test is performed: The spoofer is off, and then it is switched on around 27 s with a strong power, namely −130 dBm. Figure 12 reports the results for GPS L1 and Galileo E1B, achieved with the sets of parameters S1 and S6 in Table 3, respectively, in terms of the number of acquired satellites and estimated Doppler. As visible, the snapshot acquisition correctly detects the spoofing signal after it has been switched on, as expected.

## 6. Computational Load Analysis

A software profiling analysis of both RFI detectors has been performed to reveal the heaviest functions and to provide indications useful for reducing the complexity.

The RFI detection software module is launched in profiling mode, thereby reading a file of raw samples previously grabbed and enabling the execution of additional software functions code-implemented to measure the profiling metrics. The profiling metrics are evaluated directly in the software code, exploiting the access to system-wide clocks and high-resolution CPU timers provided by the time.h library. Such a procedure allows us to perform the profiling analysis with a very precise and accurate approach [75]. Table 4 summarizes the main hardware features of the two platforms considered for this analysis: the Adalm-Pluto and a standard PC, used as references. The RFI detectors software module, including the two detectors configured as in Table 2 and Table 3, has been executed iterating several times on both platforms, for a total of about 24 h of equivalent running time for each considered set of values.

The software profiling, in terms of the average execution time and call rate, of the main functions of the two RFI detectors is shown in Table 5 and Table 6. The GoF is configured as in Table 2, with a calibration phase performed every 10 s, thus updating the reference histogram averaging over the last 10 histograms in order to provide sufficient statistics for the profiling. The snapshot acquisition has been evaluated for the sets of parameters S1 and S6 in Table 3, respectively, for the GPS and Galileo. The analysis has been performed for a sampling rate $f_{s}=5\;Msps$ and repeated for $f_{s}=20\;Msps$, i.e., the minimum required for the FE to efficiently elaborate on the L5/E5 band. It can be noticed how the performance dramatically degrades on Adalm-Pluto for both algorithms.

Regarding the GoF, the average execution time reported in Table 5 increases as the sampling frequency grows as expected. It is not critical since it shows to be in the order of tens of ms on the Adalm-Pluto’s processor at the maximum sampling rate considered, i.e., $f_{s}=20\;Msps$, for the two main functions, as visible in the last two columns of Table 5. Furthermore, since the GoF is independent of the GNSS bandwidth, as already explained in Section 4.2.3, two instances of the same SW module can be allocated to elaborate the two required bands, i.e., L1/E1 and L5/E5.

Regarding the snapshot acquisition, overall the effect of a higher frequency is not relevant, as visible in Table 6: results for $f_{s}=20\;Msps$ are comparable to those for $f_{s}=5\;Msps$, as expected, since the FFT size, which is the main driven factor, has been kept fixed. Apart from this minor observation, the snapshot acquisition exhibits the highest computational load, as clearly visible in Table 6. Now, on a standard, PC this is not an issue since it takes about 0.6 s and 14 s for the acquisition of the whole GPS and Galileo constellations for the maximum sampling rate, i.e., $f_{s}=20\;Msps$, and maximum number of non-coherent sums, i.e., $N_{nc}=8\;$, as visible for GPS PerformCoarseAcquisition and Galileo PerformCoarseAcquisition functions in the 4th column of Table 6. On the Adalm-Pluto, performance degrades with an approximately 42–44-fold increase in processing time, rising to about 28 s for GPS and almost 10 min for Galileo.

Table 7 reports the analysis for different values of non-coherent sums, namely $N_{nc}=\{8,\;4,\;2\}$, and $f_{s}=5\;Msps$ on the Adalm-Pluto’s processor. To ease the analysis, the results for $N_{nc}=8$ in the 3rd column are the same as in the 5th column of Table 6. The other parameters, particularly the Doppler step (667 Hz and 87 Hz, respectively, for GPS and Galileo), are exactly the same as those used for the analysis in Table 6. It can be noticed that the average execution time decreases linearly with the non-coherent sum reduction, as expected: halving for 4 non-coherent sums, thus passing from 26.8 s to 13.5 s and from 9 min to 4 min for GPS and Galileo, respectively, and dividing by 4 for 2 non-coherent sums (7 s and 2 min for GPS and Galileo, respectively).

To complete the analysis, Table 8 reports the detailed profiling of the main subroutines of the heaviest function, namely the Galileo snapshot acquisition, for the set of parameters S6 in Table 3 and sampling rate $f_{s}=5\;Msps$. The call rate is also reported as a function of the acquisition parameters $N_{d}$, $N_{c}$, $N_{sat}$, and $N_{nc}$, which represent, respectively, the number of searched Doppler bins, code delay bins, satellites, and non-coherent sums, as detailed in Section 4.2.2. As it can be noticed, the function with the highest processing time is the SampleCompression, as expected. Indeed, the SampleCompression is in charge of compressing 1 ms of samples to fit to 4096 FFT points by means of the average operation, as illustrated in Figure 2. As such, the averaging cannot be parallelized, and the single instruction multiple data stream (SIMD) assembly cannot be used in this case. More than 80% of the time is spent executing the FFT function (14.54 μs × 619,586 = 9 s over 11 s total required by the Galileo PerformCoarseAcquisition), as shown in the last column of Table 8. Thus, to speed up the execution of the snapshot acquisition, a way to accelerate the FFT should be investigated. In this regard, reference [76] illustrates how to use an FFT IP core to accelerate the software in the Zynq-7000 AP SoC: a hardware FFT unit is included in the PL fabric, thus gaining 9.3X speed with respect to the execution on the NEON SIMD engine, thanks to the tightly coupled hardware co-processing capabilities of the Zynq-7000 AP SoC. Another option to be considered could also be to parallelize parts of the snapshot acquisition processing on more cores/processors. For instance, once the carrier is removed, all the operations executed for all satellites in the constellation, namely the FFT, code wipe-off, and IFFT, can be split over different cores. This solution requires a multicore architecture SoC board and could be evaluated in the near future.

Regarding the snapshot-acquisition-based RFI detector’s computational load on the L5/E5 band, a numerical evaluation cannot be provided since its implementation has not been carried out, as explained in Section 4.2.3. Some more considerations can be made. At first, due to the high computational complexity [70], a cold-start acquisition on the L5/E5a signal is unlikely to be adopted in double-frequency receiver processing, where a L1/E1-aided acquisition [71] is usually employed. Then, the ADRV9361-Z7035 board (with a AD9361 chipset aboard), originally selected and that will be employed in the next phase, features two RF receiving channels so that the two GNSS bandwidths, i.e., L1/E1 and L5/E5a, can be received and elaborated in parallel since they are synchronized with the same clock. According to the above points, an L5/E5a acquisition with precise L1/E1 assistance might be a viable solution. In particular, the L1/E1 Doppler and code delay information can be used to perform a Doppler and code refinement, namely a serial search (SS) for a very limited number of bins around the L5/E5 CAF peak estimation. The computational load of such a solution is expected to be smaller (even much smaller, depending on the number of evaluated bins) than that of the classical FFT-based PCS algorithm currently adopted in the snapshot-acquisition-based RFI detector. In this regard, reference [75] shows a 7.5-fold reduction of the average execution time for the SS on one dimension (either Doppler or code) with respect to the PCS for GPS signal.

## 7. Discussion

Although the testing experiments described above have to be considered preliminary, a discussion trying to compare the achieved performance against literature references and target key indicators, if any, is worth reporting.

Overall, the GoF turns out to be a very good solution for the detection of non-GNSS-like interferences. Indeed, it shows very good detection performance, with a sensitivity of −100 dBm on the GPS L1/Galileo E1 band and very low computational complexity even on a board with reduced computational resources like the Adalm-Pluto, with an average execution time in the order of tens of ms. Additionally, two instances of the same SW module can be used for the GPS L5/Galileo E5a band, thanks to its direct compatibility with another GNSS bandwidth. Focusing on the sensitivity from [38] and considering false alarm and detection probabilities, respectively equal to $P_{FA}=10^{-5}$ and $P_{D}=0.95$, for jamming signals, a minimum interfering power of about −110 dBm can be derived as an indicative target. Compared to this and considering that theoretically the GoF is able to reach up to −107 dBm jamming power [56], the achieved performance is not so far and can be improved with a fine adjustment of the algorithm parameters. A slight improvement is also expected with the final FE configuration (higher sampling frequency and receiving bandwidth), which will also allow us to evaluate the detection performance on the GPS L5/Galileo E5a band.

Regarding the snapshot acquisition, the detection performance on the L1/E1 band can be considered fair, with a sensitivity at 95% detection probability in the range −139.3–−137.8 dBm for GPS and 135.8–−134.3 dBm for Galileo. The computational load on the Adalm-Pluto is low for GPS L1 (28 s average execution time) but huge for Galileo L1 (almost 10 min average execution time) due to the very high number of Doppler bins to visit. Regarding the sensitivity, although a specific target has not been defined yet, it is true that a good high-sensitivity GNSS receiver can acquire signals even lower than −155 dBm. For instance, the GNSS receiver developed in [77] for space applications shows an acquisition sensitivity of −159 dBm (15 dB-Hz equivalent C/No). Now, it is well known that increasing the integration time (number of coherent and non-coherent accumulations) will improve the acquisition performance, but at the price of a worsening of the complexity [78]. In this regard, the high sensitivity acquisition techniques for lunar missions presented in [78] confirmed the fundamental importance of accurate Doppler-aided information for an effective reduction of the search space. Unfortunately, no aid can be exploited for our target application since no a priori knowledge of a spoofing source can be assumed and a full search space is recommended. A relevant computational load reduction, decreasing the average execution time by about 10× is expected to be achieved with an implementation optimization (using a hardware FFT unit and multi-core processing parallelization) on the final board. On top of this, a careful evaluation of the performance-complexity trade-off will be considered. The final board, namely the ADRV9361-Z7035 board originally selected, is expected to overcome most of the Adalm-Pluto board’s limitations in terms of computational resources, number of RF receiving channels, maximum RF bandwidth, and sampling frequency supported. This will allow us to fully support the L5/E5a bandwidth, namely to target the required FE configuration (RF bandwidth B≥20 MHz double-side, sampling rate $f_{s}\geq 20\;Msps$) and to implement the L5/E5a snapshot acquisition. Regarding this, the proposed solution, namely the L5/E5a acquisition technique with precise L1/E1 assistance, has two main limitations: the sensitivity is bounded by the one achieved on L1/E1 and the search is restricted. On the other side, the computational load of a cold-start L5/E5a acquisition makes it unfeasible for the limited computational power of the final platform. Additionally, to be really effective, a spoofing attack should target both GNSS bandwidth. Thus, the final choice will be carefully evaluated, also considering the most likely and effective spoofing attacks.

## 8. Conclusions and Future Work

This paper presents the development of an in-laboratory prototype for the detection of GNSS RFI to be hosted onboard a LEO satellite. After the selection of the target platform, the SW implementation of the RFI detection module is detailed, and a preliminary performance evaluation in a controlled environment is discussed.

The RFI detection SW module includes two RFI detectors: the GoF algorithm for *non-GNSS-like* interference (e.g., jamming) and the snapshot acquisition for *GNSS-like* one (e.g., spoofing and matched-spectrum jamming). It is coded in C and assembly and can be executed on Intel- and ARM-based processors. Performance evaluation in the presence of jamming and spoofing signals reveals suitable detection capability and sensitivity, as well as room to optimize the computational load, particularly for the snapshot-acquisition-based RFI detector.

Future activities include the porting of the SW module to the ADRV9361-Z7035 board originally selected and the algortithm optimization to improve the detection sensitivity and reduce the computational load, particularly for the snapshot-acquisition-based RFI detector. A more comprehensive test campaign with several types of interferences is also foreseen.

## Figures and Tables

**Table 1:** Adalm-Pluto’s RF FE configuration.

**GNSS Band**	GPS L1/Galileo E1
**Receiver LO Frequency (GHz)**	1.57542
**Sampling mode**	Baseband (IQ)
**Sampling frequency ** ***f*_*s*_ (MSps)**	6.0
**Intermediate frequency ** ***f*_*IF*_ (MHz)**	0
**Double-side Bandwidth (MHz)**	4.0
**ADC Quantization (bits)**	12
**Clock mode**	External
**AGC mode**	Manual
**AGC gain**	63 dB

**Table 2:** GoF-based RFI detector parameters used for the tests.

		Value
Snapshot parameters	Snapshot duration (s)	0.010
	Snapshot update time (s)	1
GoF parameters	Number of histogram bins	32
	Detection threshold (significance level)	0.95

**Table 3:** Snapshot acquisition-based RFI detector parameters used for the tests.

		GPS L1 Set of Values					Galileo E1 BSet of Values			
		S1	S2	S3	S4	S5	S6	S7	S8	S9
Snapshot parameters	Snapshot duration (s)	0.008	0.010	0.012	0.016	0.020	0.032		0.016	
	Snapshot update time (s)	1								
Acquisition parameters	FFT size	4096								
	False detection probability	1.0 × 10^−11^								
	Maximum Doppler Frequency (Hz)	20000								
	Doppler Frequency step (Hz)	667					87	167	87	167
	Code delay step (chip)	0.25								
	Coherent sums (code periods)	1								
	Non-coherent sums (code periods)	8	10	12	16	20	8		4	

**Table 4:** Platforms used for the software profiling analysis.

Platform	Platform 1	Platform 2 [64,65]
Board	Dell Optiplex 9010 Desktop PC	Adalm-Pluto
Processor	Intel^®^ Core^TM^ i7-3770	ARM Cortex™-A9
Base frequency of the processor	3.40 GHz	666 MHz
Cores	8	1
Memory	8 GB DDR3	512 MB DDR3L
Storage	1 TB HDD	32 MB Serial Flash
Operative System	Ubuntu 18.04.3 LTS (64 bit)	Linux (32 bits)

**Table 5:** Software profiling analysis of the GoF-based RFI detector obtained with $f_{s}=5\;Msps$ and $f_{s}=20\;Msps$ and parameters in Table 2.

	Call Rate (Hz)	Average Execution Time (µs)			
		Platform 1		Platform 2 [64,65]	
		$f_{s}=5\;Msps$	$f_{s}=20\;Msps$	$f_{s}=5\;Msps$	$f_{s}=20\;Msps$
*Histogram function LUT (Calibration phase)*	0.1	474.00	805.00	7340.00	11,155.00
*Histogram function LUT (Evaluation phase)*	1	487.95	622.27	4227.47	14,288.45

**Table 6:** Software profiling analysis of the snapshot-acquisition-based RFI detector obtained with $f_{s}=5\;Msps$ and $f_{s}=20\;Msps$, and set of parameters S1 and S6 in Table 3 for GPS and Galileo, respectively.

	Call Rate	Average Execution Time (µs)			
		Platform 1		Platform 2 [64,65]	
		$f_{s}=5\;Msps$	$f_{s}=20\;Msps$	$f_{s}=5\;Msps$	$f_{s}=20\;Msps$
*GPS PerformCoarseAcquisition*	1 every snapshot	571,499.17	636,101.09	26,791,668.06	28,459,665.13
*Galileo PerformCoarseAcquisition*		11,252,240.75	14,090,383.66	533,499,031.56	591,844,243.80

**Table 7:** Software profiling analysis of the snapshot-acquisition-based RFI detectors obtained with $f_{s}=5\;Msps$ and several values of the number of non-coherent sums, namely $N_{nc}=\{8,4,2\}$ for Platform 2 in Table 4.

	Call Rate	Average Execution Time (µs)		
		Platform 2 [64,65]		
		$N_{nc}=8$	$N_{nc}=4$	$N_{nc}=2$
*GPS PerformCoarseAcquisition*	1 every snapshot	26,791,668.06	13,487,729.39	6,948,932.56
*Galileo PerformCoarseAcquisition*		533,499,031.56	268,438,377.46	138,439,618.77

**Table 8:** Software profiling analysis of the Galileo snapshot acquisition and its subroutines, obtained with $f_{s}=5\;Msps$ and the set of parameters S6 in Table 3, performed on Platform 1 in Table 4.

		Call Rate (Times Every Snapshot)	Average Execution Time	
			(µs)	(%)
Calling function	*Galileo PerformCoarseAcquisition*	1	11,055,285.24	100
Main subroutines	GenerateCarrier	$N_{d}N_{c}+1=1384$	73.46	0.92
	GenerateSine		19.47	0.24
	CarrierRealWipeOff		45.34	0.57
	SampleCompression		234.26	2.93
	PerformFft	${2(N}_{d}N_{c}N_{sat}N_{nc}+1)=619,586$	14.54	81.49
	ArrayProductComplex	$N_{d}N_{c}N_{sat}N_{nc}+1=309,793$	1.55	4.34
	ArrayAbs2		0.80	2.24
	RightShiftUnsignedInt		0.58	1.62
	ArrayAddUnsignedInt		0.79	2.21
	ArrayMax	$N_{d}N_{c}N_{sat}=38,724$	2.29	0.80
	UpdateCoarseThreshold	1	53.79	0.00

## Data Availability

The data presented in this study are available in this published article.
